# Serious adverse events following immunization after ChAdOx1 nCov-19 vaccination in India: a single center experience

**DOI:** 10.11604/pamj.2021.40.66.29549

**Published:** 2021-09-29

**Authors:** Miteshkumar Rajaram Maurya, Renju Ravi, Libby Pushparajan

**Affiliations:** 1 Department of Clinical Pharmacology, Seth Gordhandas Sunderdas Medical College and King Edward Memorial Hospital, Mumbai, Maharashtra, India,; 2 K Velayudhan Memorial Hospital, Alappuzha, Kerala, India

**Keywords:** Pompholyx, herpes zoster, pancytopenia, adverse events, immunization

## To the editors of the Pan African Medical Journal

There is a huge challenge for low and middle income countries like India to conduct mass vaccination campaign for the coronavirus disease (COVID-19) that shelters the population in billions. Equally challenging is to conduct vaccine surveillance program by adverse event monitoring centres (AMCs) under the pharmacovigilance programme of India (PvPI). Vaccination campaign against COVID-19 disease started in India on 16^th^ of January 2021 in different rollout phases based on age group and comorbidities. The initial phase aimed at immunizing all health care workers and those aged > 60 years while in the second phase, all those aged >45 years with comorbidities were targeted, and subsequently for those without comorbidities and those aged 18 years and above. As on 21^st^ of April 2021, among 67,301 vaccination centres, approximately 129,332,142 doses (111,450,795 (86%) received first dose while 17,881,347 (13.8%) received second doses) of ChAdOx1 nCov-19 vaccine were administered in India [1]. However, adverse event following immunization (AEFI) reporting was only 0.017%. As of 13^th^ April 2021, in Mumbai, the financial capital of India, the total AEFI reported was 696, of which 651/696 (93.53%) were minor adverse events following immunization(AEFIs), 27/696 (3.87%) were severe AEFIs and 18/696 (2.58%) were serious AEFIs at 120 vaccination centres [1]. An AEFI is defined as any untoward medical occurrence following immunization and does not necessarily have a causal relationship with the use of the vaccine [2]. Adverse event following immunization can be related to vaccine product, quality defect, immunization error, immunization anxiety or maybe a co-incidental event [2]. We report three serious AEFIs following ChAdOx1 nCoV-19 (COVISHIELD™) vaccine administration reported to our AMC.

The first one was a case of ChAdOx1 nCoV-19 associated fever with transient pancytopenia in a 48-year-old elderly female with hypothyroidism for two years and was on tablet thyroxine 50 mcg. She developed high-grade fever with chills, rigor, burning sensation all over the body around six hours following vaccination. Given generalized body weakness and headache, the patient was admitted to our hospital on the 4^th^ day of vaccination for evaluation. She was managed with intravenous doses of ceftriaxone 1 gram twice daily, artesunate 120 mg twice daily, paracetamol 1 gram if fever >39°C, pantoprazole 40 mg once daily, a fixed drug combination of folic acid 0.7 mg, methylcobalamin 2500 mcg, niacinamide 12 mg and vitamin C 150 mg (Eldervit) in 100 ml normal saline and intravenous fluids. Laboratory results (Table 1) revealed pancytopenia like blood picture hemoglobin-9.7 gm/dl, leucocyte count-2800 cells/mm^3^, platelet count-1.2 lakhs cells/mm^3^. The blood counts restored to normal on day 6 of vaccination (Table 1) and the patient was discharged from the facility. Few case reports with elderly patients have been documented in the literature for COVID-19 disease induced pancytopenia [3] and there have been cases of idiopathic thrombocytopenic purpura (ITP) following mumps measles rubella (MMR), trivalent inactivated influenza vaccine (TIV), hepatitis B vaccine (HBV) and varicella vaccination. Coronavirus disease itself is known to cause immune thrombocytopenia and pancytopenia [4-6].

**Table 1:** laboratory investigations: day 2, 4, 5, 6, 7 post ChAdOx1 nCov-19 vaccination

Days post vaccination	Day 2	Day 4	Day 5	Day 6	Day 7
Haemoglobin (g/dl)	12.5	9.7	10.6	10.8	11.0
White Blood Cell count (cells/mm^3^)	3300	2800	3200	4200	4700
Platelet (lacs/mm^3^)	1.8	1.2	1.0	1.4	1.8

**Figure 1:**
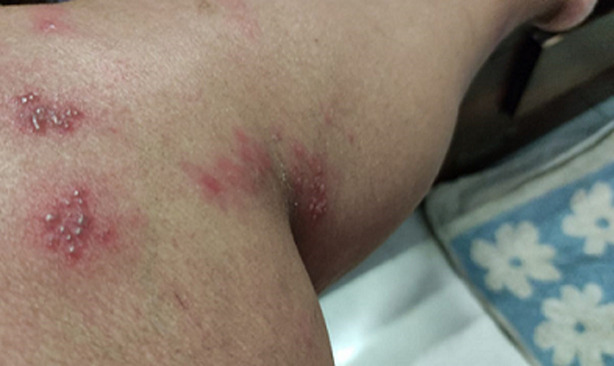
herpes zoster vesicular lesions on leg

**Figure 2:**
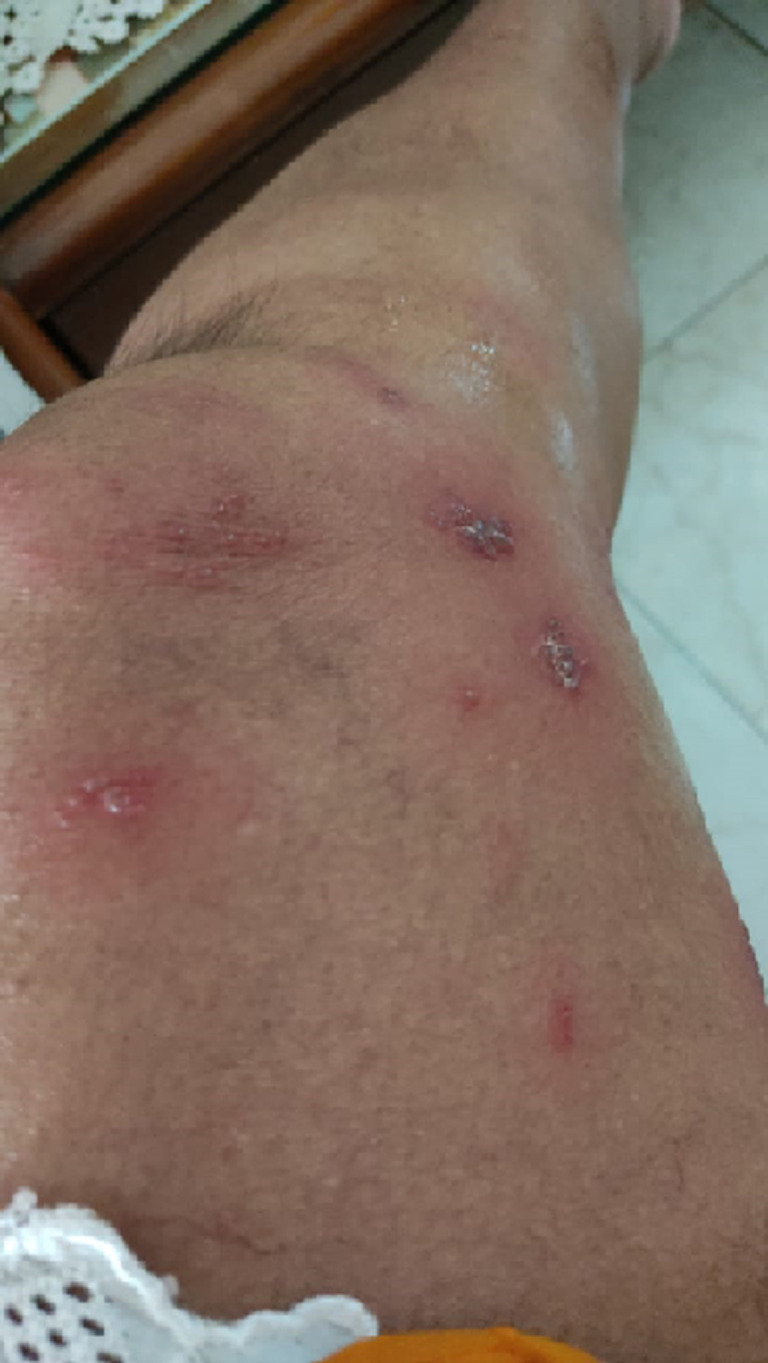
herpes zoster blisters on leg

**Figure 3:**
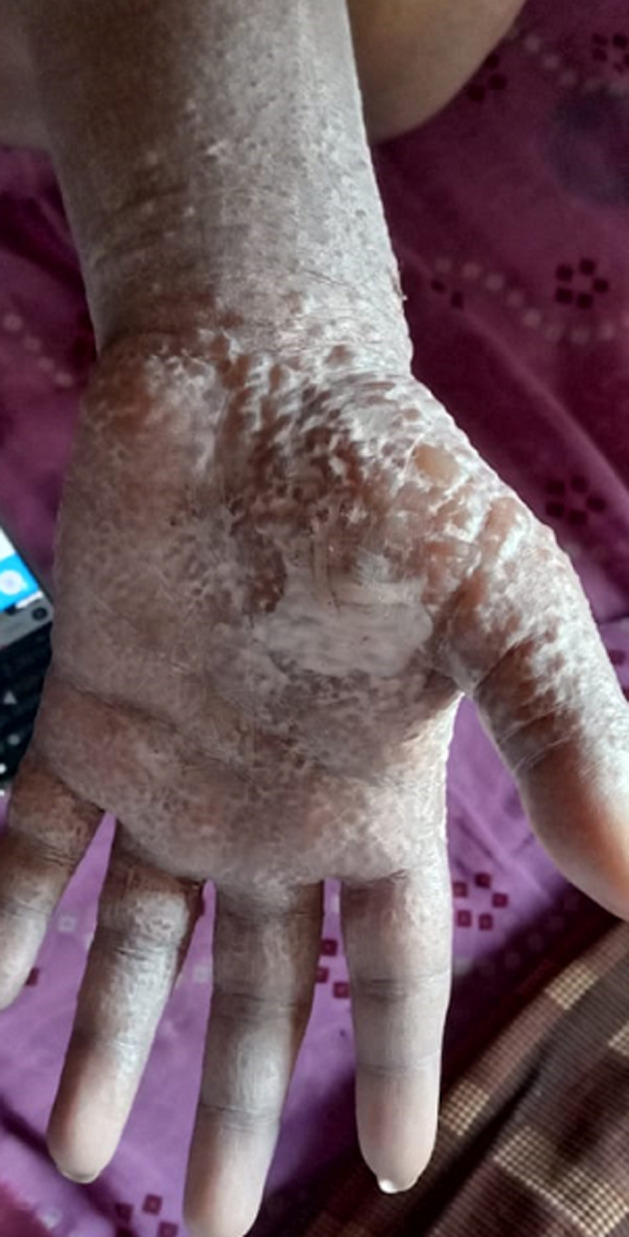
pompholyx on palm of hand

**Figure 4:**
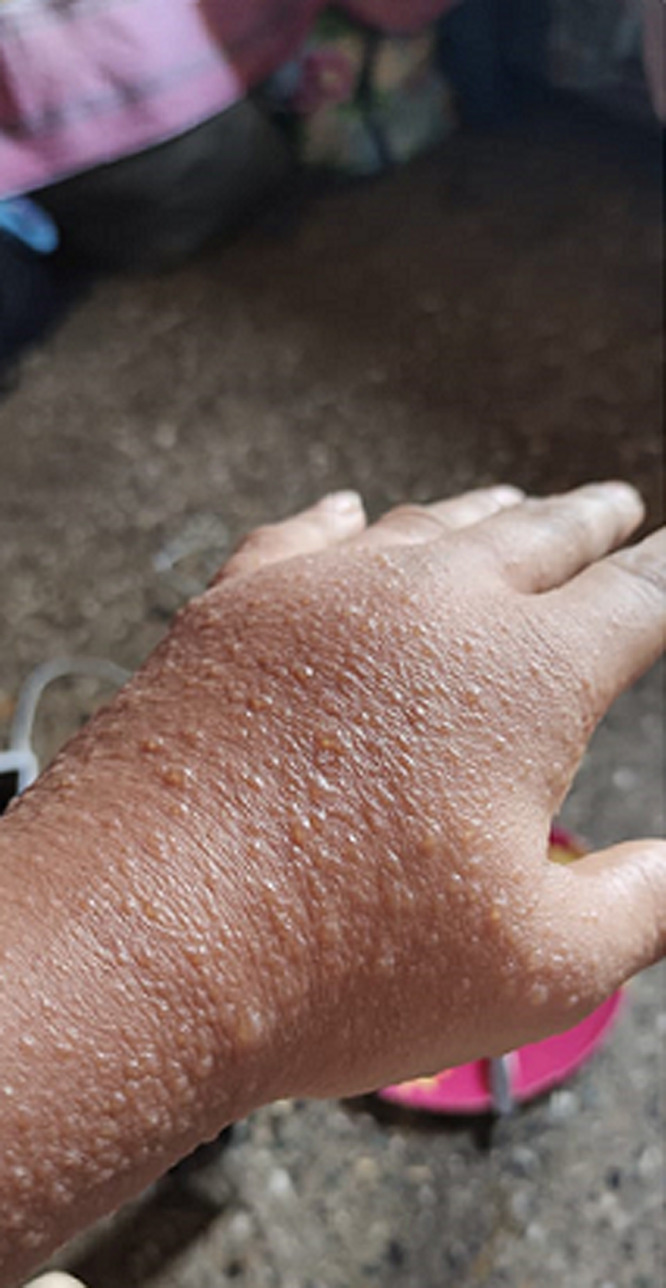
pompholyx on dorsum of hand

A 50-year-old elderly female, known hypothyroid for 15 years on tablet, levothyroxine 75 mcg daily, developed weakness all over the body with numbness over the whole left lower limb on the second day following vaccination. There was skin blister formation (Figure 1, Figure 2) and burning sensation all over the medial aspect of the left limb involving thighs and lower legs. There was no history of varicella infection in the past. A diagnosis of herpes zoster following ChAdOx1 nCov-19 vaccination was made and she was managed with tablet paracetamol 650 mg twice daily and Acyclovir 400 mg thrice daily with topical application of acyclovir ointment 5% twice daily. The herpes zoster lesions resolved on day 16 post-vaccination. Few case reports of herpes zoster eruptions, though, following administration of varicella vaccine which is live attenuated virus (Oka strain) vaccine are reported in the literature [7, 8]. COVID-19 disease itself decreases cell-mediated immunity by decreasing lymphocyte count making the patient vulnerable to herpes zoster [9]. Finally, a 30-year-old female with intradermal peeling blisters on her right hand (Figure 3, Figure 4) almost 18 hours after receiving the second dose of ChAdOx1 nCov-19 vaccine. She was diagnosed with a “pompholyx like skin lesion”. The lesions first appeared on the palm of her hands progressed to the abdomen and thighs with severe itching. She was managed with tablet, levocetirizine 10mg twice daily tablet Prednisolone 10mg twice daily and tablet. Hydroxyzine 10mg SOS with topical application of liquid paraffin, calamine (dermocalm) lotion and glycerin-olive oil soap for the skin lesions and, consequently the lesions resolved after a week. The majority of reported cases of eczematous pompholyx like reaction are with intravenous immunoglobulin [10].

## Conclusion

More safety data on the ChAdOx1 nCov-19 vaccine need to be evaluated for an association of these serious adverse events. Nevertheless, it becomes important to establish a robust vaccine surveillance system to track any adverse events following mass immunization against COVID-19.

## References

[1] Ministry of Health and Family Welfare (2021). CoWin dashboard.

[2] World Health Organisation (2018). Causality assessment of an adverse event following immunization (AEFI): user manual for the revised WHO classification.

[3] Bridwell RE, Inman BL, Birdsong S, Goss S, Long B (2021). A coronavirus disease-2019 induced pancytopenia: a case report. Am J Emerg Med.

[4] Black C, Kaye JA, Jick H (2003). MMR vaccine and idiopathic thrombocytopaenic purpura. Br J Clin Pharmacol.

[5] Ashok Shenoy K, Prabha Adhikari MR, Chakrapani M, Shenoy D, Pillai A (2001). Pancytopenia after recombinant hepatitis B vaccine--an Indian case report. Br J Haematol.

[6] Viallard JF, Boiron JM, Parrens M, Moreau JF, Ranchin V, Reiffers J (2000). Severe pancytopenia triggered by recombinant hepatitis B vaccine. Br J Haematol.

[7] Moodley A, Swanson J, Grose C, Bonthius DJ (2019). Severe herpes zoster following varicella vaccination in immunocompetent young children. J Child Neurol.

[8] Guffey DJ, Koch SB, Bomar L, Huang WW (2017). Herpes zoster following varicella vaccination in children. Cutis.

[9] Urra JM, Cabrera CM, Porras L, Ródenas I (2020). Selective CD8 cell reduction by SARS-CoV-2 is associated with a worse prognosis and systemic inflammation in COVID-19 patients. Clin Immunol.

[10] Brazzelli V, Grassi S, Savasta S, Ruffinazzi G, Carugno A, Barbaccia V (2014). Pompholyx of the hands after intravenous immunoglobulin therapy for clinically isolated syndrome: a paediatric case. Int J Immunopathol Pharmacol.

